# Phase Compensator Combined Feedback Control to Mitigate Dynamic Hysteresis in Piezoelectric Actuators

**DOI:** 10.3390/mi17091063

**Published:** 2026-09-08

**Authors:** Zhen Wang, Zekun Li, Hanqing Liu, Guanglu Hao, Bo Li, Kairui Cao

**Affiliations:** 1 The 34th Research Institute of China Electronics Technology Group Corporation, Guilin 541004, China; wangzhen3@cetc.com.cn (Z.W.); 54libolibo@163.com (B.L.); 2 School of Information and Communication Engineering, University of Electronic Science and Technology of China, Chengdu 611731, China; 3 National Key Laboratory of Laser Spatial Information, Harbin Institute of Technology, Harbin 150001, China; xtlhq2020@163.com (H.L.); guangluhao@163.com (G.H.); cao-kairui@hit.edu.cn (K.C.)

**Keywords:** piezoelectric actuator, dynamic hysteresis, phase compensator, inverse hysteresis compensation, composite control

## Abstract

The inherent dynamic hysteresis nonlinearity of piezoelectric actuators severely degrades the control accuracy of micropositioning systems. This paper proposes a composite control method based on a phase compensator and polynomial correction. Unlike conventional approaches that rely on hysteresis modeling and inversion, the proposed method equivalently treats the symmetric hysteresis of piezoelectric actuators as a phase-lag property of the system and employs a phase compensator to achieve feedforward compensation. For asymmetric hysteresis, a polynomial is cascaded with the phase compensator to correct the amplitude discrepancy between ascending and descending branches, effectively overcoming the inability of the phase compensator alone to accommodate asymmetric nonlinearity. This strategy circumvents the cumbersome procedures of precise hysteresis modeling and parameter identification, offering a simple structure, few parameters to be identified, and convenient engineering implementation within the investigated operating range. To further enhance disturbance-rejection capability and steady-state positioning accuracy, the phase–polynomial feedforward compensator is combined with PI feedback control, establishing a composite feedforward–feedback architecture for high-performance piezoelectric actuator control. Feedforward compensation and composite control experiments validate the effectiveness of the proposed method.

## 1. Introduction

**Figure 1:**
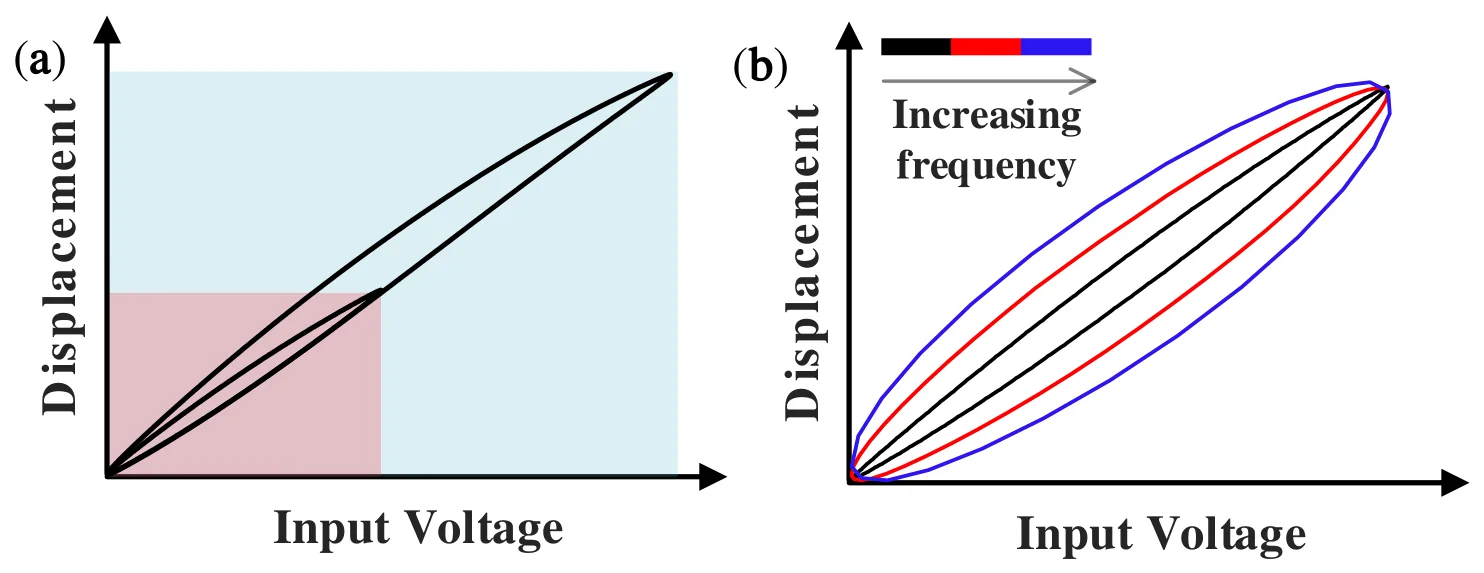
Hysteresis characteristics of piezoelectric actuators: (**a**) amplitude-dependent symmetric and asymmetric hysteresis, where the red- and blue-shaded regions denote the low- and high-amplitude excitation ranges, respectively; (**b**) frequency-dependent hysteresis with increasing excitation frequency.

Owing to their high resolution, rapid response, compact size, and high stiffness, piezoelectric actuators have been widely used in advanced applications such as ultra-precision micropositioning systems, precision optical alignment, microelectromechanical system (MEMS) fabrication, and precision measurement. They constitute one of the core actuation components in high-precision micro- and nanoscale positioning [1,2,3,4,5]. However, the inherent hysteresis of piezoelectric actuators causes the output displacement to exhibit pronounced dynamic hysteresis nonlinearity with respect to the driving voltage. The hysteresis behavior exhibits amplitude-dependent symmetry and asymmetry [6]. Specifically, symmetric hysteresis is observed under low-amplitude voltage excitation, whereas asymmetric hysteresis occurs under high-amplitude voltage excitation, as illustrated in Figure 1a. Moreover, the hysteresis behavior of piezoelectric actuators is frequency dependent, and the width of the hysteresis loop increases with the frequency of the input signal, as shown in Figure 1b. These characteristics lead to increased trajectory-tracking errors in micropositioning systems and restrict the control performance of piezoelectric actuation systems under ultra-precision operating conditions. Therefore, accurate compensation for the dynamic hysteresis nonlinearity of piezoelectric actuators is crucial for improving the control accuracy of piezoelectric micropositioning systems.

Considerable research has been conducted worldwide on compensating for the hysteresis nonlinearity of piezoelectric actuators. Existing approaches are predominantly model based, in which an accurate mathematical hysteresis model is developed to reproduce the hysteresis loops and subsequently used to design a feedforward compensation controller. Current hysteresis modeling methods can generally be classified into two categories: physics-based models and phenomenological models. Physics-based models describe macroscopic hysteresis responses by establishing electromechanically coupled constitutive equations derived from microscopic physical mechanisms, including ferroelectric domain switching and lattice-strain evolution [7,8,9]. These models have clear physical interpretations and can explain the intrinsic origin of hysteresis from a mechanistic perspective. However, their practical applicability is limited by the complexity of multiphysics coupling in piezoelectric materials, pronounced sample-to-sample variations in model parameters, and relatively poor generalization capability. In contrast, phenomenological models do not require a detailed investigation of the microscopic physical mechanisms underlying hysteresis. Instead, they establish mathematical input-output relationships directly from experimental response data. Phenomenological models can be further divided into differential-equation-based models and hysteresis-operator superposition models. The first category includes representative models such as the Bouc–Wen model [10] and the Duhem model [11]. These models describe the frequency-dependent dynamic characteristics of hysteresis using first- or higher-order differential equations and can effectively reproduce the dynamic evolution of piezoelectric hysteresis. Nevertheless, they often suffer from strong parameter coupling and considerable difficulty in parameter identification. The second category consists of superposition models based on hysteresis operators, such as the Preisach model [12], the Prandtl–Ishlinskii (PI) model [13], and the Maxwell model [14]. These models approximate complex hysteresis loops through the weighted superposition of a large number of elementary operators. Although they generally provide high modeling accuracy, they require the identification of numerous parameters and involve relatively high computational complexity.

To characterize the asymmetric hysteresis of piezoelectric actuators, extensive efforts have been devoted to modifying and improving conventional symmetric hysteresis models. By introducing asymmetric weighting functions, piecewise thresholds, and differentiated operator parameters into operator-based models, these approaches overcome the limitation of traditional models that can only represent symmetric hysteresis. Likewise, by restructuring the parameters of differential-equation-based models and incorporating piecewise nonlinear correction coefficients, accurate fitting of the ascending and descending branches of asymmetric hysteresis loops can be achieved. In addition, to capture the rate-dependent behavior of piezoelectric actuators, many researchers have incorporated the input rate into static hysteresis models or formulated the weighting and threshold coefficients as functions of the input rate [15]. Although these approaches differ in their specific formulations, most rate-dependent hysteresis models incorporate the derivative of the input signal into conventional hysteresis models. In contrast, other researchers have regarded dynamic hysteresis as the combined effect of static nonlinear hysteresis and system dynamics, and have consequently developed Hammerstein models consisting of a static hysteresis element connected in series with a linear dynamic element [16,17]. In a Hammerstein model, the static hysteresis model is independent of the input rate and describes only rate-independent hysteresis, whereas the transfer function characterizes the dynamic behavior of the system. The combined action of the static hysteresis model and the transfer function enables the dynamic hysteresis behavior of piezoelectric actuators to be effectively modeled.

In addition to conventional model-based compensation approaches, recent studies have increasingly investigated adaptive and online hysteresis-compensation strategies. Su et al. developed an event-triggered sliding-mode predictive control method for piezoelectric-actuated vibration isolation systems subject to hysteresis and time delays, incorporating online approximation and predictive control mechanisms [18]. Nie et al. proposed a hysteresis-estimator-based adaptive fuzzy control method, in which dynamic hysteresis is estimated online to improve tracking performance under system uncertainties and time-varying output constraints [19]. These advanced methods provide strong adaptability to uncertainties and varying operating conditions, but generally involve more elaborate online estimation, adaptation, or control computation. In contrast, the present work focuses on a simplified engineering-oriented compensation strategy that directly constructs a feedforward compensator from the measured phase characteristics and employs a polynomial to correct the residual asymmetric nonlinearity. Its main advantages therefore lie in structural simplicity, fewer parameters to be identified, low online computational burden, and convenient real-time implementation.

This paper proposes a hysteresis-compensation strategy that does not rely on hysteresis models from the perspective of equivalent representation of hysteretic nonlinearity. Based on the similarity between symmetric hysteresis loops and the phase-lag loops of linear systems, the symmetric hysteresis of piezoelectric actuators is equivalently represented as a phase-lag characteristic, and a phase compensator is employed to achieve feedforward compensation. To address the nonlinear and asymmetric hysteresis of piezoelectric actuators, a composite correction structure consisting of a phase compensator cascaded with a polynomial is further proposed. The polynomial is used to correct the output signal of the phase compensator, thereby compensating for asymmetric hysteresis. Compared with conventional compensation methods based on hysteresis models, the proposed approach offers the advantages of fewer parameters, a simpler structure, and easier engineering implementation. The main contributions of this paper are summarized as follows:

1. A phase-compensator-based feedforward control method is proposed. By exploiting the morphological similarity between symmetric hysteresis loops and the phase-lag loops of linear systems, the symmetric hysteresis under low-amplitude excitation is equivalently represented as the phase-lag behavior of a linear system. The phase compensator is designed to account for both the equivalent phase lag induced by hysteresis and the frequency-dependent dynamic lag at different operating frequencies, with the aim of compensating dynamic hysteresis over a relatively wide frequency range.
2. For the asymmetric hysteresis occurring under high-amplitude excitation, a cascaded correction structure combining the phase compensator and a polynomial is proposed. The polynomial performs amplitude correction on the output of the phase compensator to overcome the limitation of the phase compensator alone in compensating asymmetric hysteresis.
3. To further reduce the residual error of feedforward compensation and improve disturbance rejection and steady-state control accuracy, the proposed phase–polynomial feedforward compensator is combined with PI feedback control to form a composite feedforward–feedback control scheme, with the aim of achieving high-precision suppression of symmetric and asymmetric dynamic hysteresis nonlinearities of piezoelectric actuators over a broad range of operating conditions.

The remainder of this paper is organized as follows. Section 2 introduces the existing asymmetric hysteresis modeling method based on the Madelung rules. Section 3 analyzes the similarity between symmetric hysteresis and the phase-lag loop of a linear system, and then presents the design principle of the phase compensator as well as the proposed feedforward compensation methods for symmetric and asymmetric hysteresis. Section 4 further develops a composite control scheme consisting of the phase–polynomial feedforward compensator and PI feedback control. Section 5 describes the experimental platform and validates the effectiveness of the proposed methods through feedforward compensation and composite control experiments. Finally, Section 6 concludes the paper.

## 2. Hysteresis-Model-Based Feedforward Compensation

In [20], we proposed a hysteresis model based on the Madelung rules to characterize both the symmetric and asymmetric hysteresis behaviors of piezoelectric actuators. The model consists of two components: (1) an erasure mechanism and (2) a description of the hysteresis curves. The erasure mechanism enables the model to operate in real time in accordance with the Madelung rules. The hysteresis-curve description characterizes the ascending and descending branches, as well as the shape information of the major and minor hysteresis loops.

**Figure 2:**
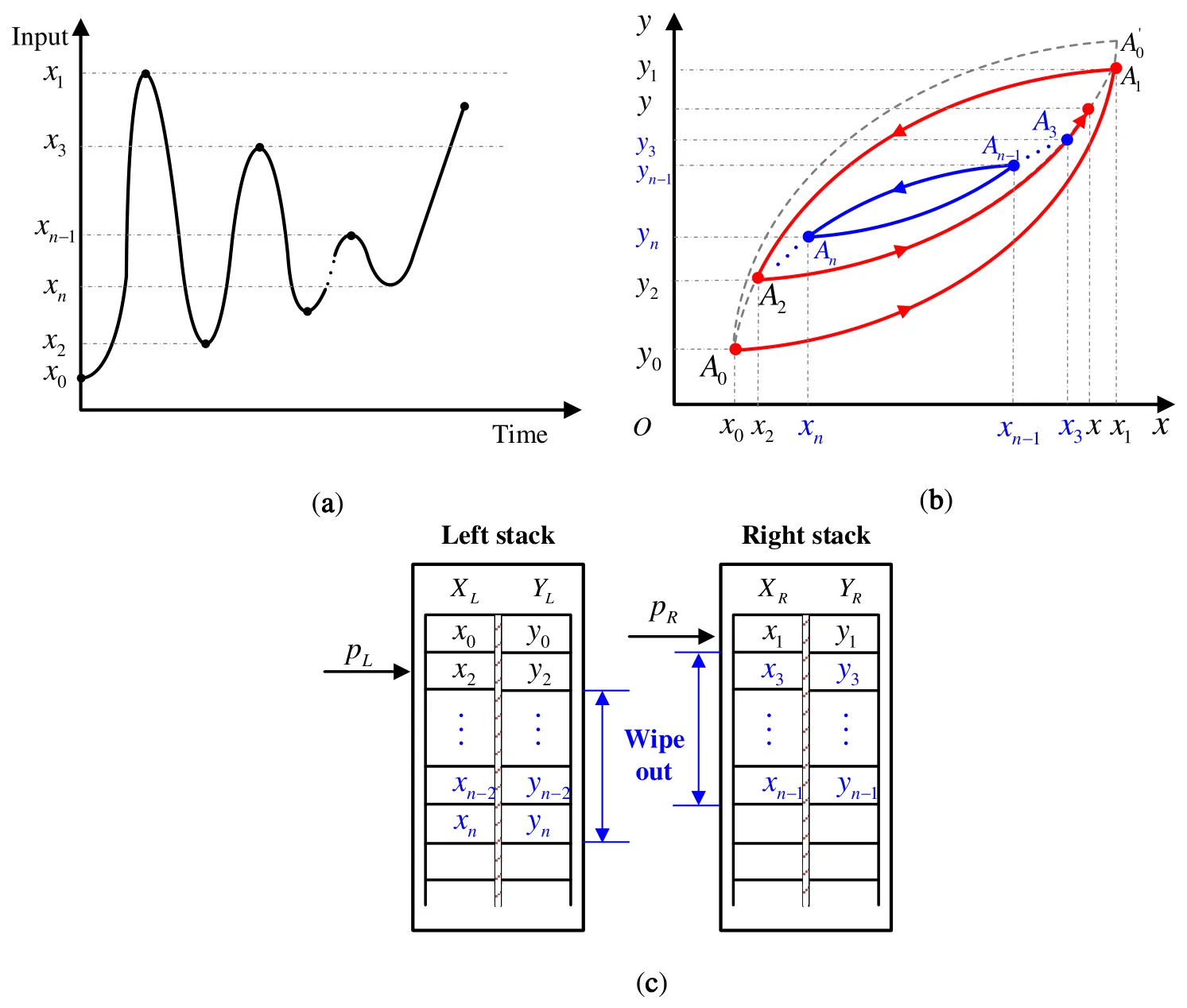
Erasure mechanism based on the Madelung rules: (**a**) input signal and sequentially generated turning points; (**b**) corresponding turning points on the hysteresis loop; and (**c**) storage and wiping-out of historical turning points in the left and right stacks. The red curves indicate the major hysteresis branches, whereas the blue curves and symbols indicate the minor hysteresis branch and the associated turning-point information.

According to the Madelung rules, the hysteresis response depends on the current input direction and the historical turning points. When the input reverses its direction, a new turning point is recorded. If the input exceeds a previous extremum, the corresponding obsolete turning-point information is erased, thereby preserving the memory and wiping-out properties of the hysteresis model. The principle of the turning-point erasure mechanism is illustrated in Figure 2, while detailed implementation procedures are available in our previous work [20].

The hysteresis curves are characterized through curve fitting. When polynomial functions are employed to describe the curve shapes, the ascending and descending branches of the major hysteresis loop can be expressed, respectively, as follows:

(1) $$f_{0}\left(x\right)=\sum_{i=1}^{n=5}p_{i}x^{i}$$

(2) $$g_{0}\left(x\right)=\sum_{i=1}^{n=5}q_{i}x^{i}$$

**Figure 3:**
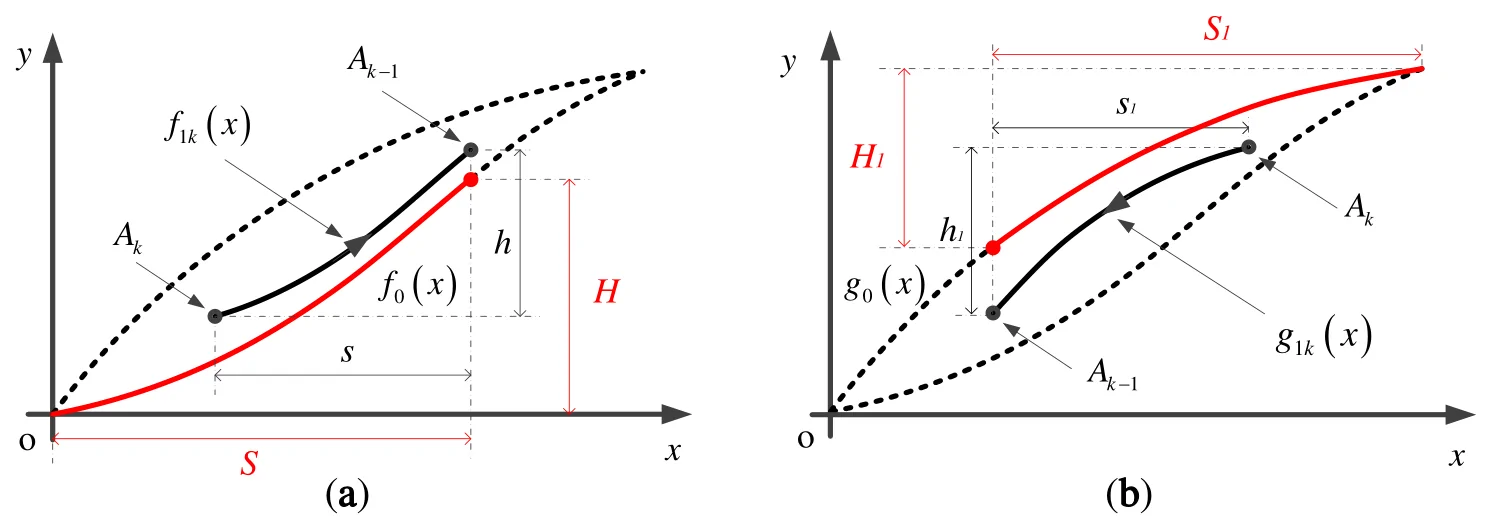
Description of the hysteresis curves: (**a**) scaling relationship for an ascending minor hysteresis branch; (**b**) scaling relationship for a descending minor hysteresis branch.

As shown in Figure 3, the minor hysteresis loop is obtained by scaling the red segment of the major hysteresis loop in the *x*- and *y*-directions.

In summary, the major-loop branch functions $f_{0}\left(u\right)$ and $g_{0}\left(u\right)$, together with the turning-point memory and wiping-out rules, provide the basis for describing both major and minor hysteresis loops. It should be noted that $f_{0}\left(u\right)$ = $g_{0}\left(u\right)$ indicates that the major ascending and descending branches have identical shapes, in which case the asymmetric hysteresis model reduces to a symmetric hysteresis model. For feedforward compensation, a hysteresis model is first established using the measured input–output data, after which the corresponding inverse hysteresis model is obtained. Finally, the inverse hysteresis model is cascaded with the system, and its output is applied as the input to the system, thereby compensating for the hysteresis effect.

The Madelung-rule-based hysteresis model used here is essentially rate-independent and is therefore more suitable for low-frequency conditions. As the excitation frequency increases, rate-dependent effects become significant, making accurate modeling and parameter identification more difficult. Its applicability may be further limited in complex actuator structures by additional nonlinearities caused by structural compliance, preload, amplification mechanisms, and hinge friction. These limitations motivate the phase-compensation strategy adopted in this work.

## 3. Phase-Compensator-Based Feedforward Compensation

### 3.1. Comparison Between Symmetric Hysteresis and Phase-Lag Loops

Under low-amplitude voltage excitation, piezoelectric actuators exhibit symmetric hysteretic nonlinearity, whose loop shape is similar to the phase-lag loop of a linear system at a given frequency. In the following, without considering the underlying physical mechanism, an illustrative example is presented to demonstrate the similarity between symmetric hysteresis and a phase-lag loop. A low-amplitude periodic signal with a frequency of 1Hz, u(t)=Asin(2πft−π/2)+A is applied to the piezoelectric actuator. The input voltage signal u(t) and the corresponding displacement response $y_{1}\left(t\right)$ are recorded. Here, *H* denotes the symmetric hysteresis exhibited by the piezoelectric actuator under low-amplitude voltage excitation.

**Figure 4:**
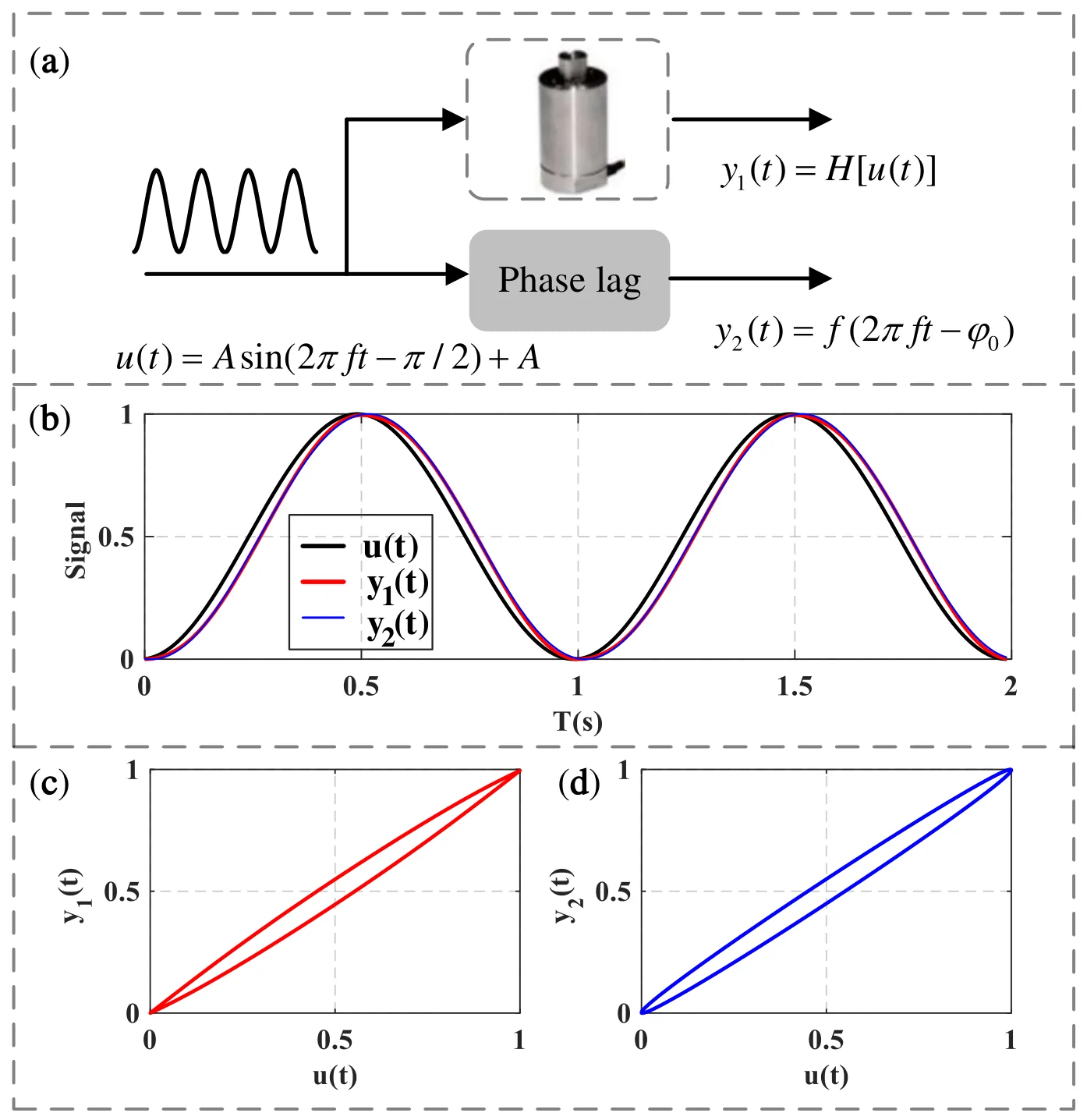
Comparison between symmetric hysteresis and an equivalent phase-lag loop: (**a**) schematic of the hysteresis response and the corresponding phase-lag representation; (**b**) normalized time-domain signals u(t), $y_{1}\left(t\right)$, and $y_{2}\left(t\right)$; (**c**) symmetric hysteresis loop of the piezoelectric actuator; and (**d**) phase-lag loop of the equivalent linear system. The black, red, and blue curves denote u(t), $y_{1}\left(t\right)$, and $y_{2}\left(t\right)$, respectively.

Meanwhile, the signal $y_{2}\left(t\right)$ is obtained by delaying u(t) by a certain time interval, such that $y_{2}\left(t\right)$ lags behind u(t) by a phase angle $\varphi_{0}$, as shown in Figure 4a. For a clearer comparison, the signals u(t), $y_{1}\left(t\right)$, and $y_{2}\left(t\right)$ are normalized such that their amplitudes are scaled to the same range. The processed signals are shown in Figure 4b.

It can be observed that, although u(t) and $y_{1}\left(t\right)$ have the same amplitude, a distinct discrepancy exists between them owing to the hysteresis effect of the piezoelectric actuator. When $\varphi_{0}$ is assigned an appropriate fixed value, $y_{1}\left(t\right)$ and $y_{2}\left(t\right)$, although not completely identical, exhibit a high degree of similarity, as illustrated in Figure 4b.

Figure 4c,d show the symmetric hysteresis loop of the piezoelectric actuator and the phase-lag loop of the linear system, respectively. A clear similarity can be observed between the two loops. Based on this observation, the symmetric hysteresis of the piezoelectric actuator under low-amplitude voltage excitation is equivalently represented in this study by a linear system with an initial phase lag of $\varphi_{0}$. It should be noted that the above equivalent representation is motivated by the observed similarity under sinusoidal excitation and is used in this study as an engineering approximation rather than a rigorous mathematical equivalence. The phase-compensation operation itself is not restricted to sinusoidal reference signals. To further examine its applicability to non-sinusoidal inputs, additional triangular-wave experiments are conducted in Section 5.3. The present experimental validation is limited to the frequency range of 10–100 Hz, corresponding to the target operating range considered in this work. Further theoretical analysis and experimental validation at higher frequencies and with more complex reference trajectories remain topics for future study.

### 3.2. Compensation for Symmetric Hysteresis of Piezoelectric Actuators

The dynamic hysteresis of a piezoelectric actuator can be decomposed into the combined effects of static hysteretic nonlinearity and linear dynamic lag. The former originates from the inherent hysteresis characteristic H of the piezoelectric material and is independent of the input frequency. When a low-frequency signal is applied to the piezoelectric actuator, the dynamic effects of the piezoelectric system are negligible, and only static hysteresis is observed. In this case, the static hysteresis can be equivalently represented as a linear system with a constant phase lag, which can be expressed as:

(3) $$\phi \left(j\omega \right)=\phi_{0}$$

where ω=2πf denotes the angular frequency. It should be noted that this phase lag is constant and does not vary with the input-signal frequency, which is consistent with the rate-independent characteristic of static hysteresis.

The second component is the linear dynamic behavior G(s) arising from the mechanical lag and electromechanical coupling inertia inherent in the piezoelectric actuator. This component exhibits typical frequency dependence, with the phase shift between the input and output becoming increasingly pronounced as the excitation frequency increases. The actual phase lag associated with this component is given by

(4) $$\varphi_{2}\left(j\omega \right)=∠G\left(j\omega \right)$$

Therefore, the dynamic hysteresis behavior of the piezoelectric actuator can be regarded as that of a linear system with an initial phase lag. Its magnitude–frequency and phase–frequency characteristics can be expressed as

(5) $$A_{G}\left(j\omega \right)=\left|G(j\omega )\right|$$

(6) $$\varphi_{G}\left(j\omega \right)=\varphi_{1}\left(j\omega \right)+\varphi_{2}\left(j\omega \right)=\varphi_{0}+∠G\left(j\omega \right)$$

From the perspective of linear frequency-domain analysis, a proportional–derivative (PD) element inherently provides phase-lead characteristics. The proportional term provides only magnitude gain without introducing any phase shift, whereas the derivative term differentiates the input signal and thereby generates a phase lead. When the PD element is incorporated into the system as a compensator, the phase lead generated by its derivative term can offset the inherent phase lag of the controlled plant. By appropriately selecting the proportional and derivative coefficients, a specified phase correction can be achieved at a particular frequency. The PD feedforward controller can be expressed as

(7) $$C_{1}\left(s\right)=k_{1}+k_{2}s$$

where s=jω denotes the Laplace variable, and $k_{1}$ and $k_{2}$ are the proportional and derivative coefficients of the feedforward controller, respectively. The magnitude and phase characteristics introduced by this feedforward controller are given by

(8) $$A_{C_{1}}\left(j\omega \right)=\left|C_{1}\left(j\omega \right)\right|=\sqrt{k_{1}^{2}+k_{2}^{2}\omega^{2}}$$

(9) $$\varphi_{C_{1}}\left(j\omega \right)=∠C_{1}\left(j\omega \right)=\tan^{-1}\left(\frac{k_{2}\omega }{k_{1}}\right)$$

It can be observed from the above equations that, by appropriately adjusting $k_{1}$ and $k_{2}$, the following condition can be satisfied while maintaining $A_{C_{1}}\left(j\omega \right)\approx 1$:

(10) $$\left|\varphi_{C_{1}}\left(j\omega \right)\right|=\varphi_{0}+∠G\left(j\omega \right).$$

Consequently, the dynamic hysteresis of the piezoelectric actuator can be compensated at the frequency f=ω/(2π). It should be noted that the controller $C_{1}\left(s\right)$ must be designed for a specific input frequency. When the input frequency changes, the values of $k_{1}$ and $k_{2}$ in $C_{1}\left(s\right)$ must also be readjusted to achieve satisfactory compensation performance. Moreover, when the input signal contains multiple frequency components, the controller cannot effectively compensate for the signal.

Because the fixed-parameter compensation element $C_{1}\left(s\right)$ can achieve ideal phase cancellation only at a single frequency, it cannot satisfy the requirements for high-precision compensation under broadband excitation. To uniformly compensate for the frequency-dependent phase lags occurring at different frequencies within the target operating bandwidth, the parameters of the phase compensator must be specifically designed.

By appropriately configuring the adjustable parameters of the compensation element, its phase-lead characteristic can be made to cover the required phase-compensation range, thereby simultaneously suppressing dynamic phase deviations over a relatively wide frequency range. The phase compensator can be designed in the following form:

(11) $$C_{2}\left(s\right)=\frac{b_{m}s^{m}+b_{m-1}s^{m-1}+\cdots +b_{1}s+b_{0}}{a_{n}s^{n}+a_{n-1}s^{n-1}+\cdots +a_{1}s+a_{0}}$$

where $a_{n}$ and $b_{m}$ denote the denominator and numerator coefficients of the phase compensator $C_{2}\left(s\right)$, respectively.

The parameters of the phase compensator are identified using the least-squares method. Subject to the constraint imposed by the magnitude–frequency response, the minimization of the input–output phase error of the piezoelectric actuator system is taken as the optimization objective. The unknown coefficients of the phase compensator are determined through offline fitting using a least-squares algorithm, enabling the compensator to generate a phase-lead correction that matches the hysteresis-induced phase lag over the target operating frequency band. The objective function is formulated as follows:

(12) $$L\left(a_{n},b_{m}\right)=\sum_{i=1}^{N}\left\{{\left[\varphi_{G}\left(j\omega_{i}\right)-∠C_{2}\left(j\omega_{i};a_{n},b_{m}\right)\right]}^{2}+{\left[\left|C_{2}\left(j\omega_{i};a_{n},b_{m}\right)\right|-1\right]}^{2}\right\}$$

**Figure 5:**
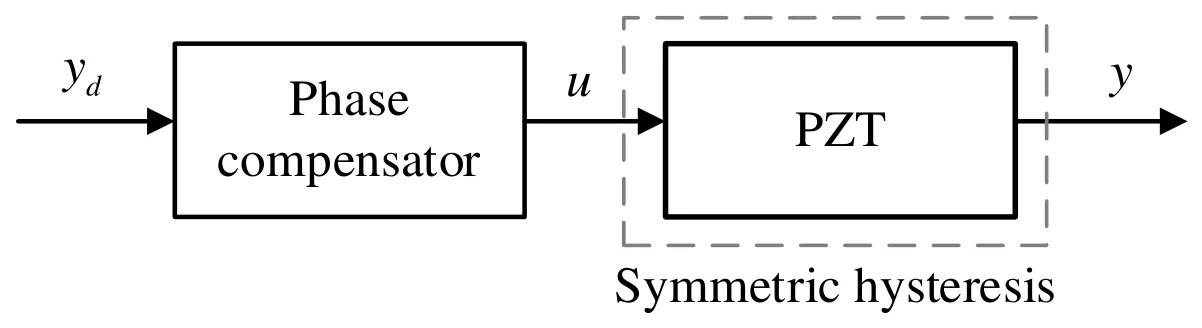
Schematic diagram of the phase-compensator-based method for symmetric hysteresis compensation.

Under low-amplitude excitation, the ascending and descending displacement responses of the piezoelectric actuator are nearly identical. Under this operating condition, the designed phase compensator is cascaded with the system to offset the dynamic hysteresis through phase lead, thereby achieving compensation for symmetric hysteresis, as illustrated in Figure 5.

### 3.3. Compensation for Asymmetric Hysteresis of Piezoelectric Actuators

**Figure 6:**
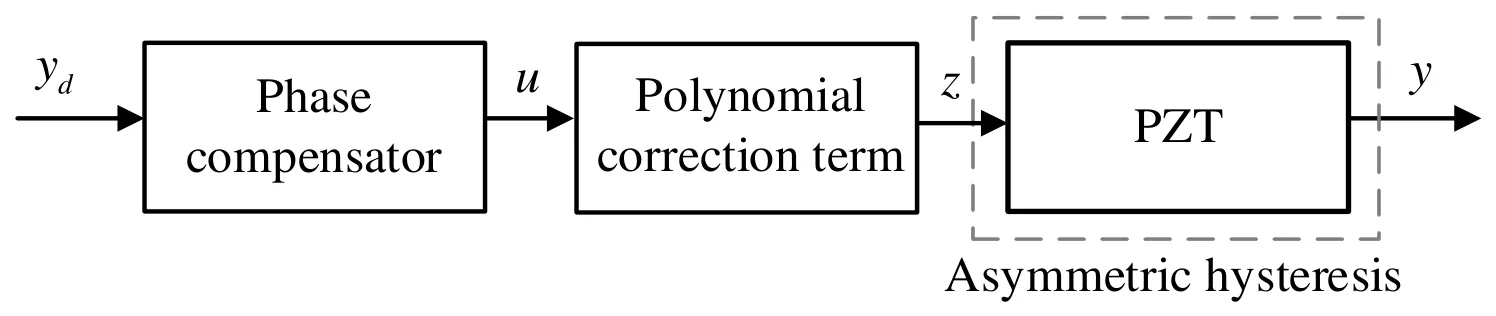
Schematic diagram of the phase-compensator–polynomial cascade method for asymmetric hysteresis compensation.

When a piezoelectric actuator is subjected to high-amplitude driving-voltage excitation, a pronounced discrepancy arises between the displacement responses during the ascending and descending strokes, resulting in a typically asymmetric hysteresis loop. Existing studies have proposed composite structures in which a symmetric hysteresis model is cascaded with a polynomial to characterize asymmetric hysteresis behavior [21,22,23]. By exploiting the polynomial’s capability to correct the amplitude of the model output, these structures enable accurate representation of the asymmetric hysteresis profile. Inspired by this hierarchical cascade-correction strategy, this study combines the identified phase compensator in series with a polynomial correction element. The phase compensator first counteracts the phase lag associated with dynamic hysteresis, after which the polynomial corrects the amplitude discrepancy between the ascending and descending strokes. In this manner, compensation for the asymmetric dynamic hysteresis of the piezoelectric actuator is achieved, as illustrated in Figure 6. The polynomial can be expressed as:

(13) $$z\left(u\right)=\sum_{i=1}^{n=5}c_{i}u^{i}$$

It should be emphasized that the polynomial in Equation (13) is a single-valued static nonlinear mapping and does not itself generate the two branches of the hysteresis loop. The distinction between the ascending and descending branches originates from the preceding phase-compensation process. Owing to the phase-dependent trajectory, the ascending and descending strokes corresponding to the same original driving-voltage level generally produce different instantaneous outputs from the preceding compensation stage. Consequently, the inputs applied to the polynomial correction element are different for the two strokes. The higher-order nonlinear terms therefore produce different amplitude corrections, which modify the curvature, slope, and relative spacing of the two branches and reduce the residual asymmetry. In other words, the polynomial reshapes the output of the preceding compensation stage rather than independently reproducing the multivalued hysteresis behavior. Similar cascade-based nonlinear correction strategies have also been employed for asymmetric hysteresis modeling and compensation [21,22,23].

The fifth-order polynomial is selected to balance fitting accuracy and model simplicity. A polynomial of excessively low order provides insufficient degrees of freedom to characterize the residual asymmetric amplitude discrepancy between the ascending and descending branches, resulting in underfitting. In contrast, further increasing the polynomial order provides only limited improvement in compensation accuracy while introducing redundant parameters, additional computational burden, and a higher risk of overfitting. It should also be noted that the polynomial is employed only to correct the residual local asymmetric nonlinearity after phase compensation, rather than to model the entire hysteresis loop. Therefore, the nonlinear fitting burden imposed on the polynomial is relatively limited. Furthermore, its generalization capability is supported by experiments under additional excitation conditions that were not used for parameter identification, in which stable compensation performance is maintained.

## 4. Composite Control Based on the Phase Compensator

**Figure 7:**
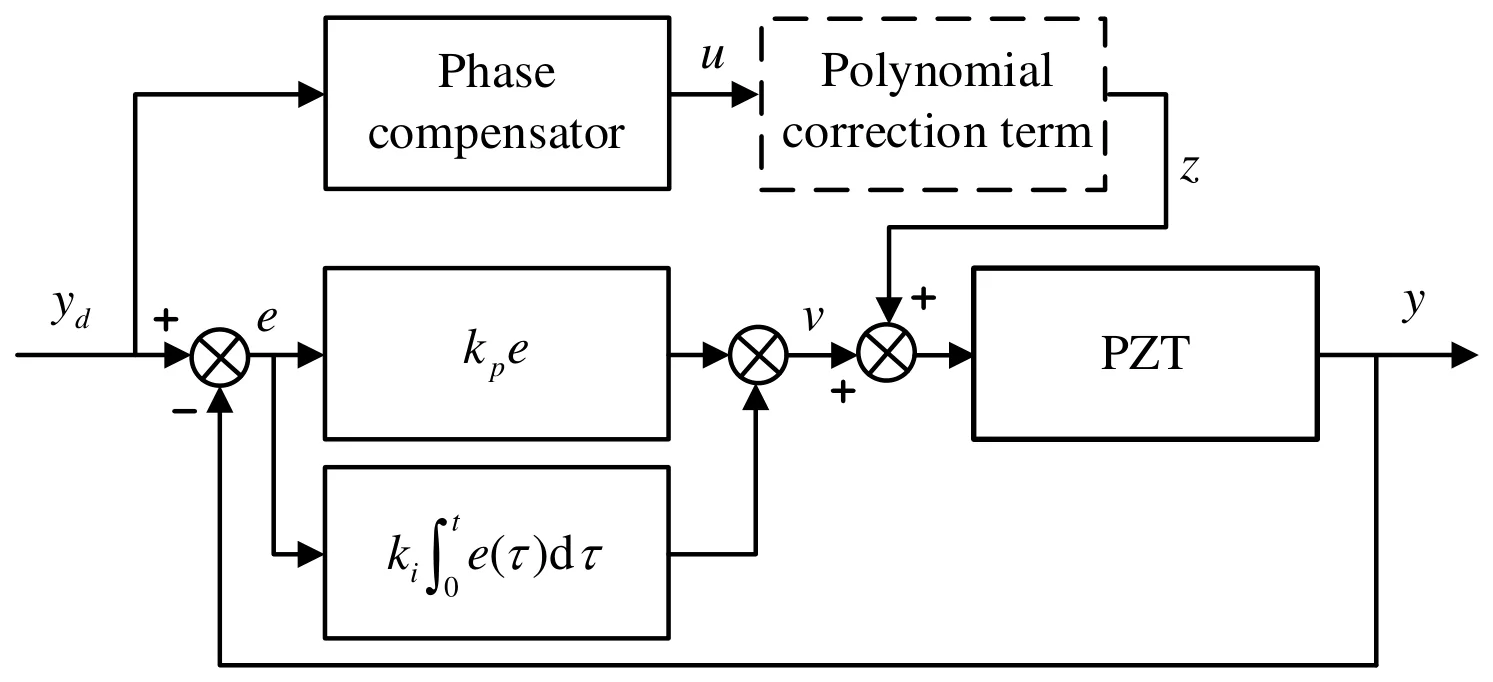
Schematic diagram of the composite feedforward–feedback control scheme based on the phase compensator.

Although the feedforward branch composed of a phase compensator cascaded with a polynomial can substantially reduce the tracking errors induced by the dynamic hysteresis of the piezoelectric actuator, dynamic hysteresis and phase lag are not strictly equivalent. Therefore, the proposed feedforward compensation method inevitably leaves a certain amount of residual error. Moreover, owing to parameter-identification errors and external disturbances, feedforward compensation alone cannot guarantee satisfactory trajectory-tracking accuracy. To address these limitations, feedback control is introduced to form a composite control architecture. The feedback loop corrects the residual errors remaining after feedforward compensation in real time, thereby enhancing the disturbance-rejection capability and control accuracy of the system. The phase-compensator-based composite control scheme is illustrated in Figure 7. In this study, a PI controller is adopted, and its output can be expressed as:

(14) $$v=k_{p}e+k_{i}\int_{0}^{t}e\;dt$$

where $e=y_{d}-y$ denotes the tracking error, and $k_{p}$ and $k_{i}$ are the proportional and integral gains of the PI controller, respectively.

In the experiments, the PI controller is implemented in an incremental discrete form as

(15) $$u\left(k\right)=u\left(k-1\right)+k_{p}\left[e\left(k\right)-e\left(k-1\right)\right]+k_{i}T_{s}e\left(k\right),$$

where $e\left(k\right)=y_{d}\left(k\right)-y\left(k\right)$ is the tracking error at the *k*th sampling instant.

## 5. Experiments

### 5.1. Experimental Platform

**Figure 8:**
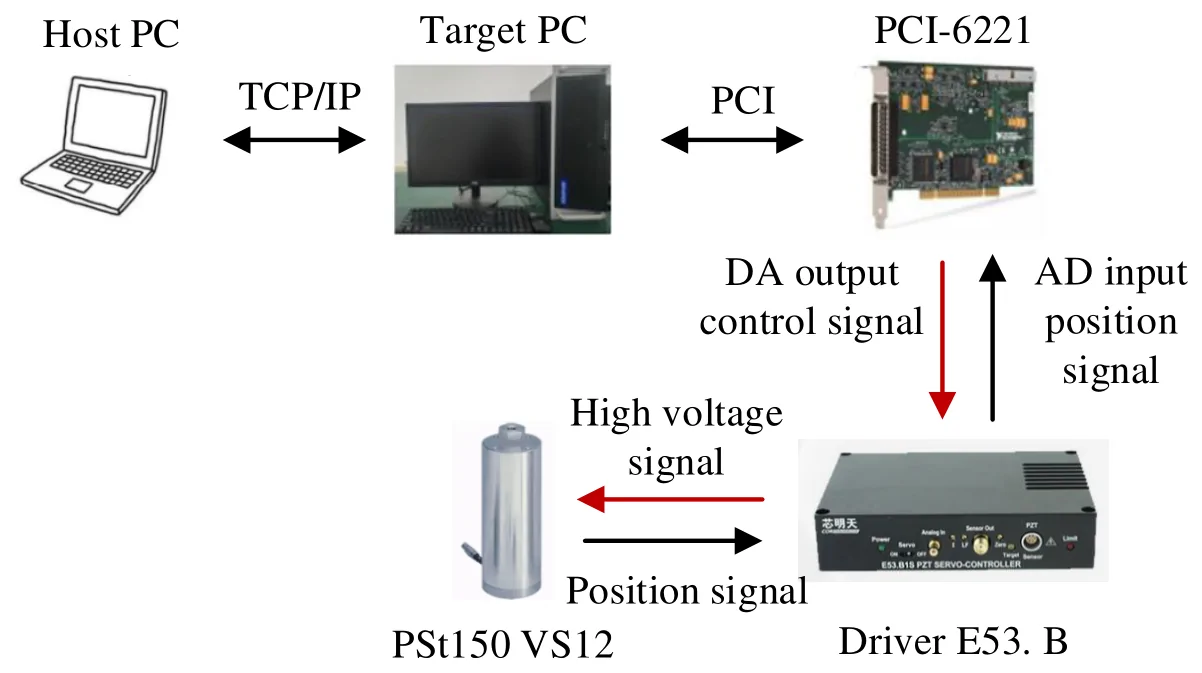
Schematic diagram of the experimental platform.

The experimental platform consists of a piezoelectric actuator, a high-voltage amplifier, a data acquisition and control card, a host computer, and a real-time target computer, as shown in Figure 8. The piezoelectric actuator is a PSt150/7/20 VS12S manufactured by Harbin Core Tomorrow Science & Technology Co., Ltd. (Harbin, China). A resistive strain gauge sensor (SGS) is bonded to the actuator to measure and provide real-time feedback of its actual output displacement. The actuator has a nominal travel of 19 μm at 0–150 V and a capacitance of 1.8 μF; the integrated SGS has a measurement range of 0–19 μm, a linearity of 0.1% F.S., and a repeatability of 0.05% F.S. The high-voltage amplifier is used to amplify the control and feedback signals. The PCI-6221 data acquisition and control card, manufactured by National Instruments (Austin, TX, USA), provides 16-bit analog-to-digital and digital-to-analog conversion. To satisfy the real-time control requirements of the experimental platform, LabVIEW 2016 with the Real-Time Module is employed to provide a real-time control and data acquisition environment. The feedforward compensation and control algorithms are compiled on the host computer using LabVIEW modules and then downloaded to the target computer. The algorithms can be modified in the block diagram and executed to conduct experiments and acquire experimental data. The sampling period of the experimental platform is set to 200 μs. All experiments were conducted under unloaded conditions, with displacement ranges of approximately 0–6.5 μm and 0–18.5 μm for the low- and high-amplitude cases, respectively.

### 5.2. Parameter Identification Results

First, under low-amplitude voltage excitation, the values of $k_{1}$ and $k_{2}$ in the PD controller are sequentially adjusted at different frequency points $\omega_{i}$(i=1,2,3,…,n) to provide the required phase lead $\varphi_{C_{1}}\left(j\omega_{i}\right)$. This phase lead compensates for the system phase lag $\varphi_{G}\left(j\omega_{i}\right)$, thereby linearizing the input–output relationship of the system.

Next, the frequency-domain relationships given in Equations (8) and (9) are used to calculate the phase $\varphi_{C_{1}}\left(j\omega_{i}\right)$ of the PD controller at each frequency point. At this stage, the following relationship holds:

(16) $$\varphi_{G}\left(j\omega_{i}\right)=-\varphi_{C_{1}}\left(j\omega_{i}\right).$$

It should be noted that the phase terms in Equations (10), (12) and (16) correspond to different stages of the compensation design. In Equation (10), $\varphi_{C_{1}}\left(j\omega \right)$ denotes the phase lead provided by the PD controller, whereas $\varphi_{0}$ and ∠G(jω) describe the phase lag associated with hysteresis and the linear dynamic component. The PD controller is used here only as a frequency-domain measurement tool to determine the phase compensation required at individual frequency points, rather than as the final feedforward compensator. Equation (12) uses the phase information obtained at multiple frequencies to identify the broadband phase compensator $C_{2}\left(s\right)$, while Equation (16) expresses the corresponding phase-cancellation relationship during parameter identification. Therefore, although the phase terms appear in different forms, they describe different stages of the same compensation procedure and are logically consistent.

**Figure 9:**
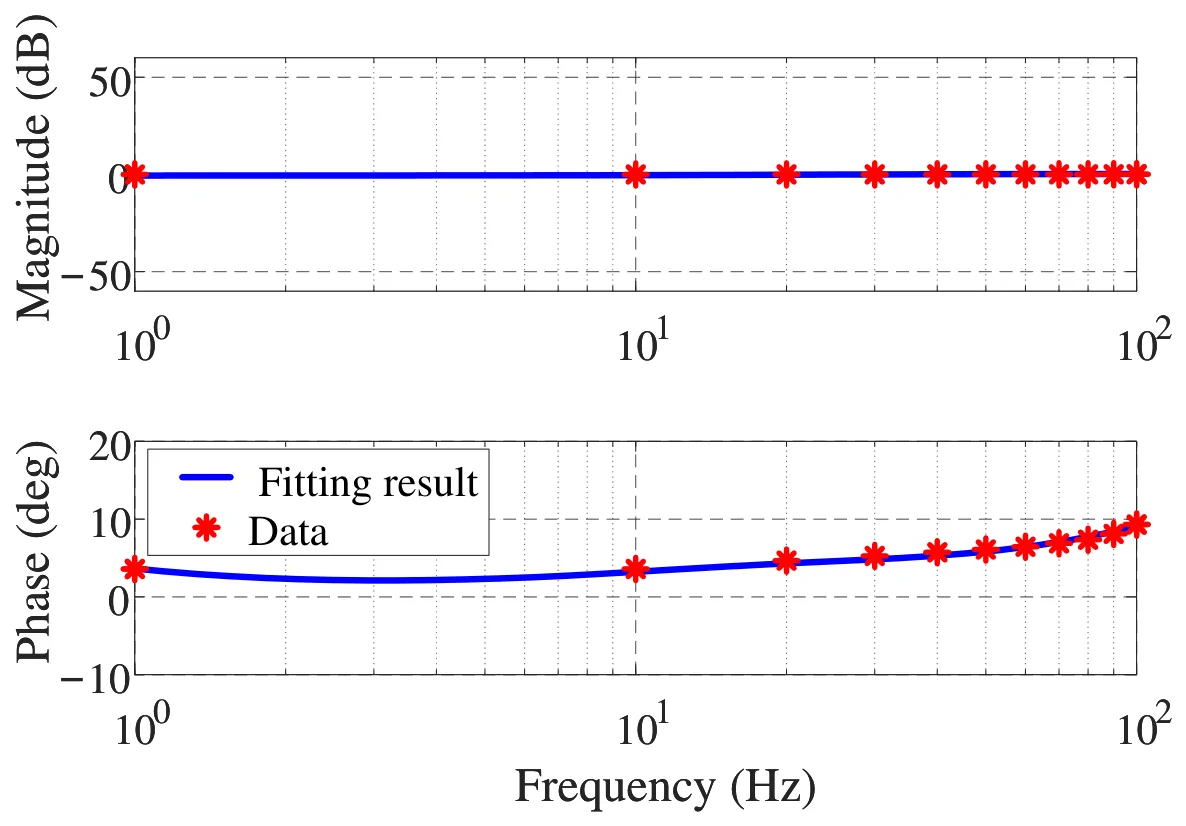
Identification results of the phase compensator.

These data are then globally fitted using the least-squares method by minimizing the objective function given in Equation (12). The fitting results of the phase compensator are shown in Figure 9, and the resulting expression of the phase compensator is given in Equation (17).

(17) $$C_{2}\left(s\right)=\frac{2.27\times 10^{-3}s^{3}+3.46s^{2}+1.33\times 10^{3}s+7.08\times 10^{3}}{s^{2}+1.27\times 10^{3}s+7.55\times 10^{3}}$$

Although the identified compensator in Equation (17) has a third-order numerator and a second-order denominator, this continuous-time transfer function is not directly discretized in the digital implementation. Instead, the third-order numerator is decomposed into a derivative term and a remaining proper term. The derivative term is discretized using the relation $s=\left(z-1\right)/\left(T_{s}z\right)$, whereas the remaining term is discretized using the zero-order-hold method. The two discrete components are then combined to obtain the implementable discrete phase compensator:

(18) $$C_{2}\left(z\right)=\frac{11.93z^{3}-32.42z^{2}+29.3z-8.804}{z^{3}-1.775z^{2}+0.7757z}.$$

The sampling period is $T_{s}=0.0002\;s$, corresponding to a sampling frequency of 5 kHz. The stability of the discrete implementation was verified from its pole distribution, with all poles located inside the unit circle. In addition, because the target compensation range is 10–100 Hz and high-frequency measurement noise is suppressed by the hardware low-pass filtering of the measurement channel, the high-frequency gain introduced by the derivative term remains controllable within the investigated frequency range. Therefore, no additional digital derivative filter is employed.

A high-amplitude sinusoidal signal is applied to the piezoelectric actuator to capture the shape characteristics of the asymmetric hysteresis loop. The polynomial correction term is then identified using the least-squares method based on the output of the piezoelectric actuator and that of the phase compensator. The identified polynomial is given in Equation (19).

(19) $$z\left(u\right)=0.1646u^{5}-0.3866u^{4}+0.5353u^{3}-0.3218u^{2}+1.008u$$

To quantitatively evaluate the effectiveness of the proposed feedforward compensation and composite control methods, the normalized maximum error $e_{\mathrm{NME}}$ and the normalized root-mean-square error $e_{\mathrm{NRMSE}}$ are adopted as performance metrics. They are defined as follows:

(20) $$e_{\mathrm{NME}}=\frac{\max_{i}\left|y_{d_{i}}-y_{i}\right|}{\max_{i}\left(y_{i}\right)-\min_{i}\left(y_{i}\right)}\times 100\%$$

(21) $$e_{\mathrm{NRMSE}}=\frac{\sqrt{\frac{1}{M}\sum_{i=1}^{M}{\left(y_{d_{i}}-y_{i}\right)}^{2}}}{\max_{i}\left(y_{i}\right)-\min_{i}\left(y_{i}\right)}\times 100\%$$

### 5.3. Feedforward Compensation Experiments

**Figure 10:**
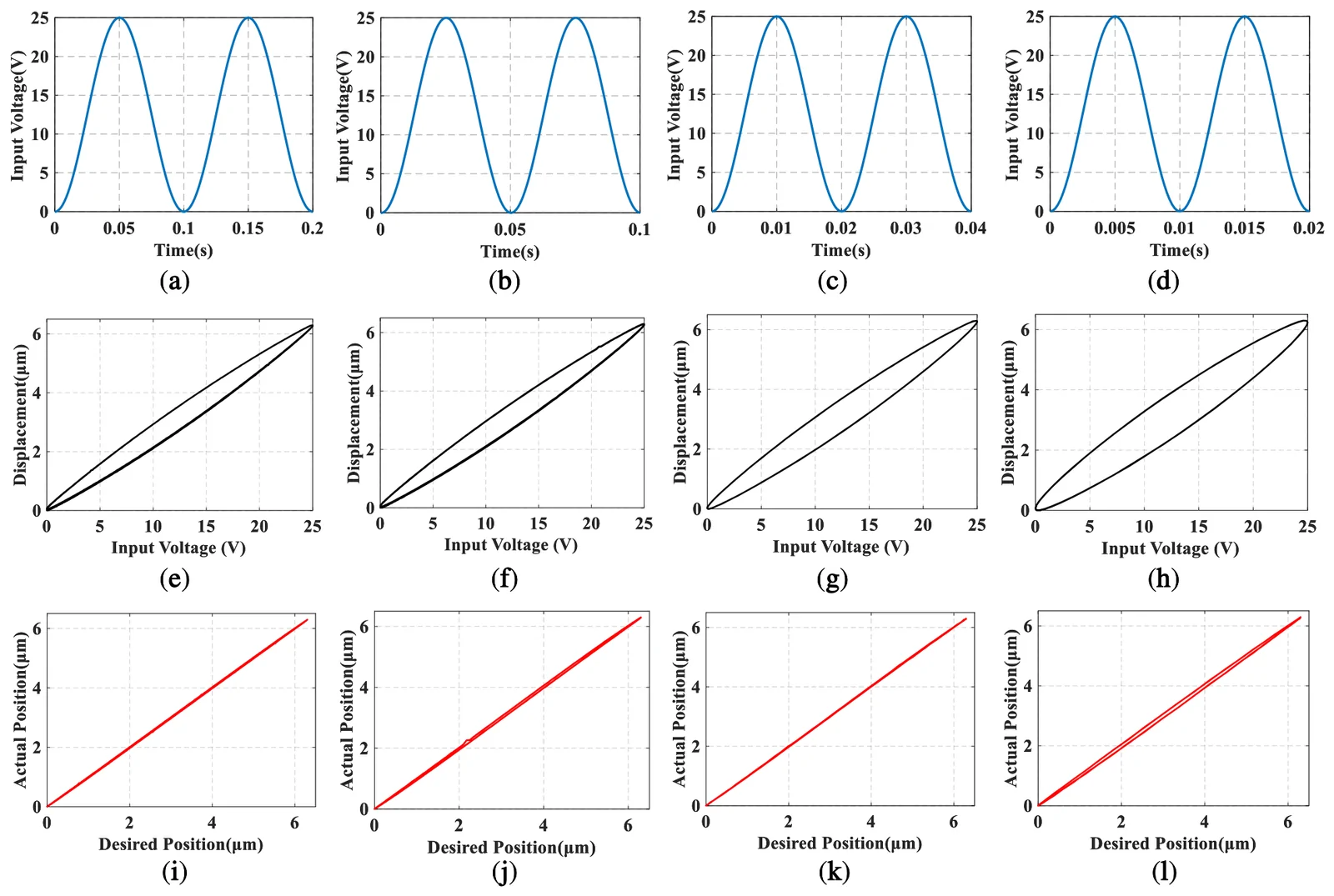
Symmetric hysteresis compensation under sinusoidal excitation at 10, 20, 50, and 100 Hz: (**a**–**d**) input voltage signals; (**e**–**h**) uncompensated hysteresis loops; and (**i**–**l**) compensated desired–actual displacement relationships.

Figure 10a–d show the sinusoidal input voltage signals at 10, 20, 50, and 100 Hz, respectively, while Figure 10e–h present the corresponding uncompensated input voltage–output displacement hysteresis loops of the piezoelectric actuator at different excitation frequencies without compensation. It can be clearly observed that the actuator exhibits pronounced frequency-dependent symmetric dynamic hysteresis. As the frequency of the driving signal gradually increases, the width of the hysteresis loop continuously expands, the phase lag between the input and output becomes increasingly severe, and the nonlinear distortion grows more evident. After feedforward compensation is implemented using the phase compensator designed in this paper, the corresponding desired–actual displacement relationships are shown in Figure 10i–l. Compared with the original hysteresis characteristics, it can be seen that the phase-lead compensation effectively offsets the errors caused by the dynamic hysteretic response. At all tested frequencies, the actual output displacement closely tracks the desired displacement curve, and the hysteresis loop is significantly compressed. These experimental results demonstrate that, for the symmetric dynamic hysteresis of piezoelectric actuators under low-amplitude excitation, the phase-compensator-based feedforward strategy alone can achieve satisfactory suppression of dynamic hysteresis and can significantly reduce the positioning error induced by dynamic hysteresis.

**Figure 11:**
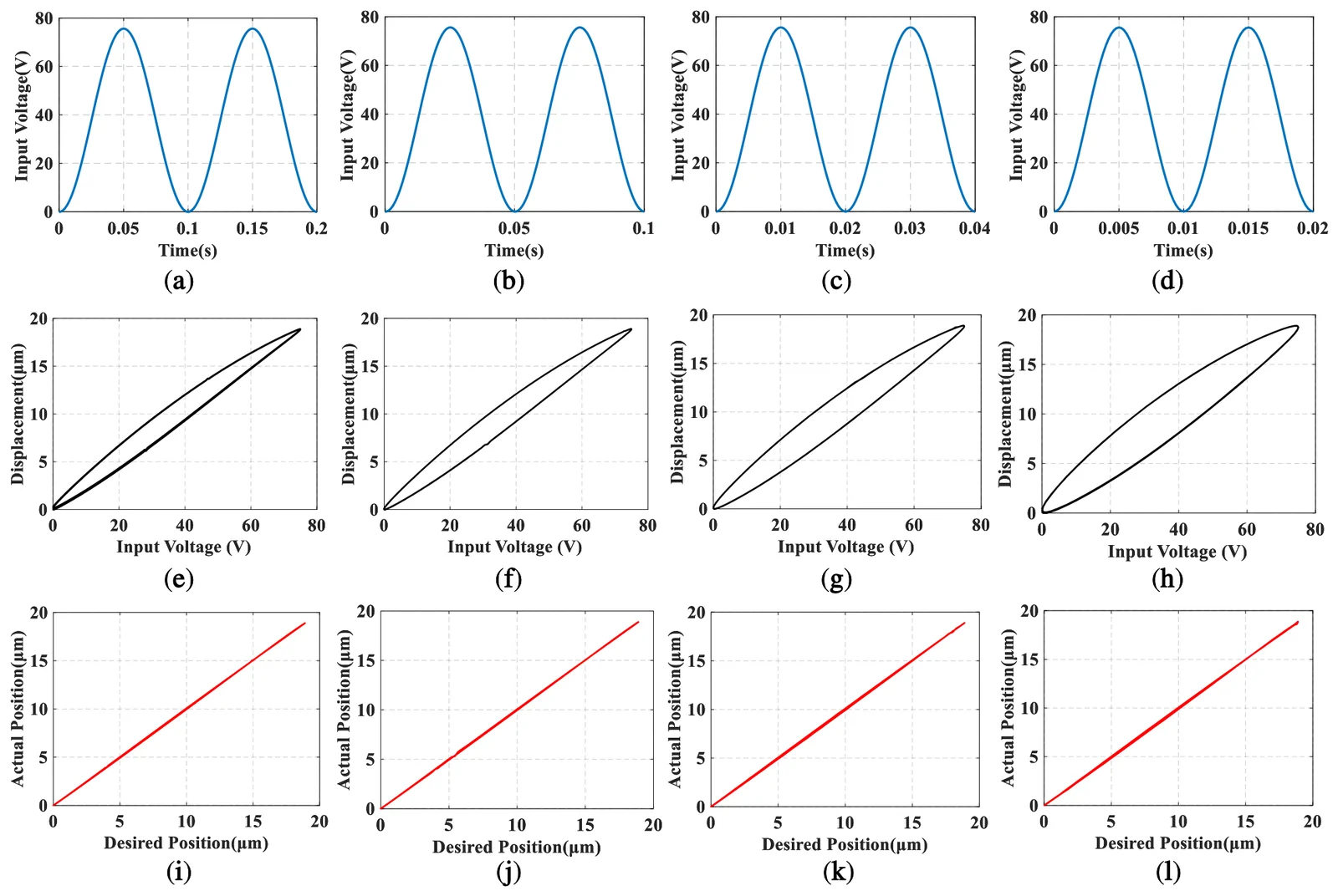
Asymmetric hysteresis compensation under sinusoidal excitation at 10, 20, 50, and 100 Hz: (**a**–**d**) input voltage signals; (**e**–**h**) uncompensated hysteresis loops; and (**i**–**l**) compensated desired–actual displacement relationships.

Under high-amplitude excitation, the hysteresis of the piezoelectric actuator arises from the coupling of linear dynamic phase lag and static asymmetric amplitude distortion. Figure 11 presents the corresponding results under high-amplitude sinusoidal excitation, where (a–d) show the input voltage signals, (e–h) show the uncompensated asymmetric hysteresis loops, and (i–l) show the compensated desired–actual displacement relationships at 10, 20, 50, and 100 Hz, respectively. Therefore, a single phase compensator alone is insufficient to correct the asymmetric amplitude error. In the cascaded feedforward structure constructed on the basis of polynomial series correction, the phase compensator is used to offset the frequency-varying phase lag, and the polynomial fitting is used to correct the asymmetric amplitude deviation. The experimental curves show that, after compensation with this scheme, the hysteretic nonlinearity is effectively suppressed at different frequencies, and the actual positioning output exhibits excellent linearity. This confirms that the proposed method can effectively compensate for the asymmetric dynamic hysteresis characteristics of piezoelectric actuators.

The hysteresis loops and displacement-tracking curves presented above provide only a qualitative assessment of the hysteresis-suppression performance. To further quantify the compensation accuracy, the values of $e_{\mathrm{NME}}$ and $e_{\mathrm{NRMSE}}$ after symmetric and asymmetric hysteresis compensation are calculated at four excitation frequencies: 10, 20, 50, and 100Hz. The statistical results are summarized in Table 1.

A comparison of the two operating conditions shows that the phase compensator alone can reduce the tracking error associated with symmetric hysteresis to a very low level. After the polynomial amplitude-correction element is introduced, compensation for asymmetric hysteresis is also achieved. The proposed method maintains low $e_{\mathrm{NME}}$ and $e_{\mathrm{NRMSE}}$ values under both symmetric and asymmetric hysteresis conditions over the tested frequency range. In particular, the asymmetric-hysteresis case does not exhibit a systematic increase in compensation error compared with the symmetric case, indicating that the polynomial correction effectively reduces the residual amplitude nonlinearity introduced by hysteresis asymmetry.

**Figure 12:**
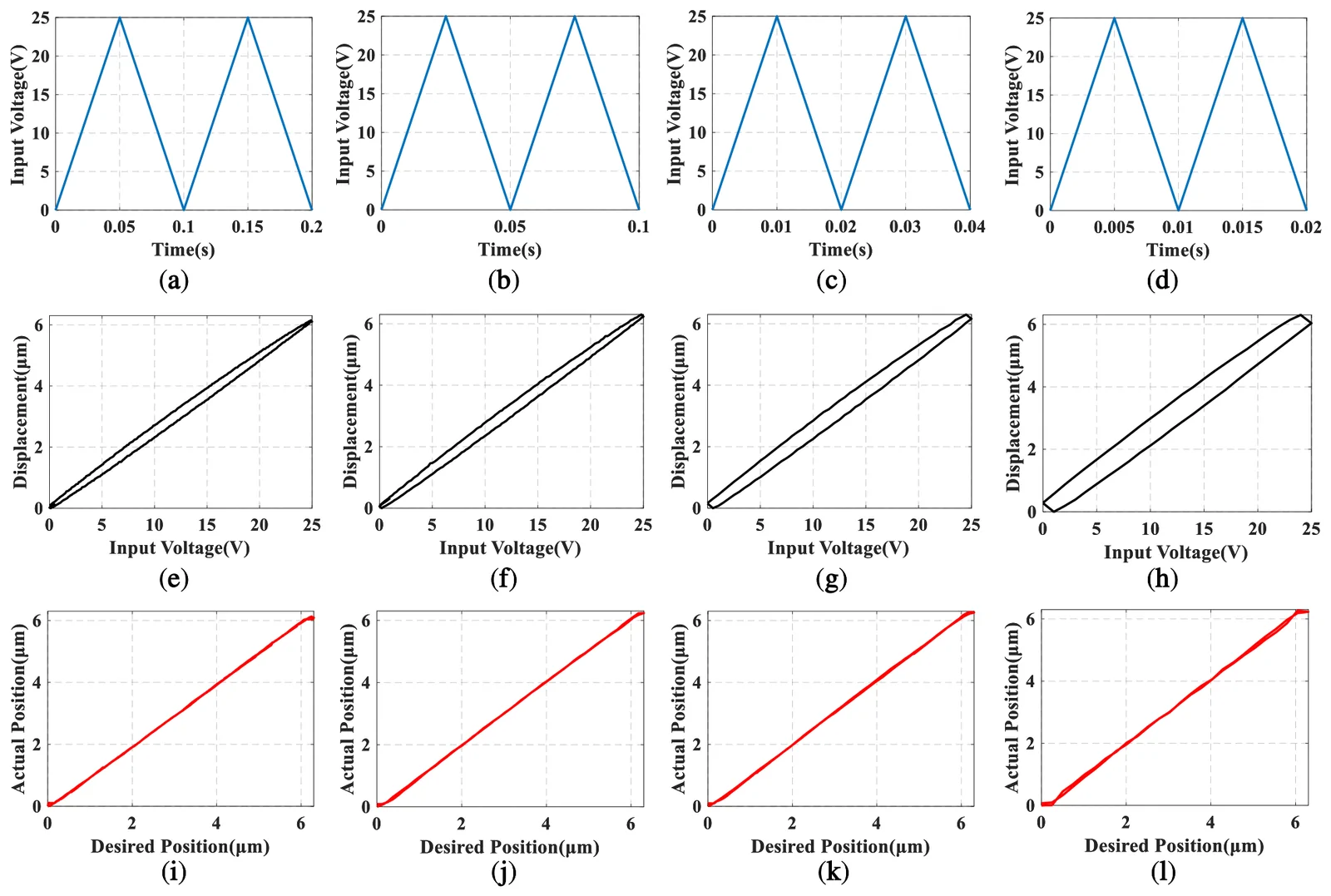
Symmetric hysteresis compensation under triangular-wave excitation at 10, 20, 50, and 100 Hz: (**a**–**d**) input voltage signals; (**e**–**h**) uncompensated hysteresis loops; and (**i**–**l**) compensated desired–actual displacement relationships.

**Figure 13:**
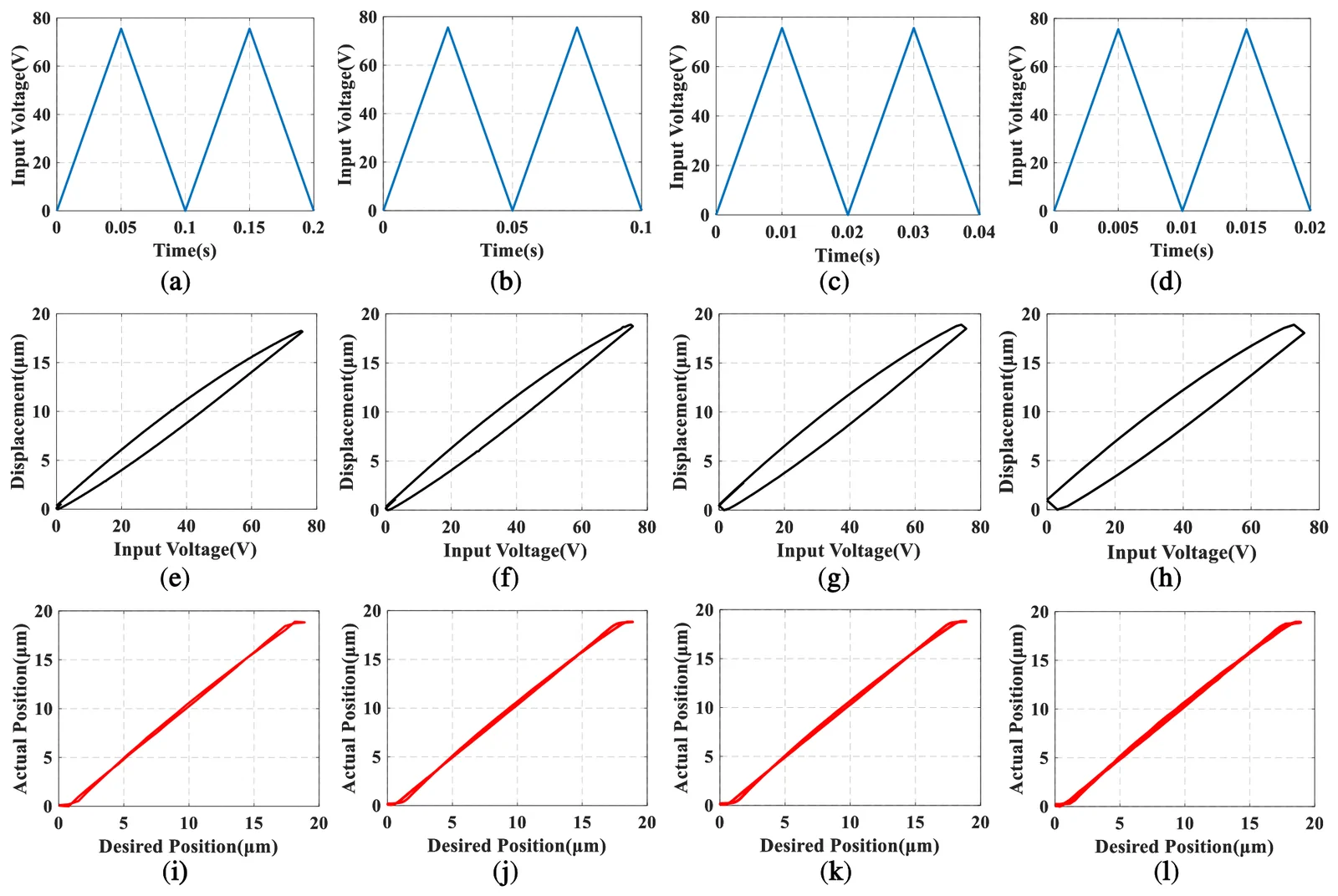
Asymmetric hysteresis compensation under triangular-wave excitation at 10, 20, 50, and 100 Hz: (**a**–**d**) input voltage signals; (**e**–**h**) uncompensated hysteresis loops; and (**i**–**l**) compensated desired–actual displacement relationships.

To further evaluate the applicability of the proposed feedforward compensation method to non-sinusoidal inputs, additional experiments were conducted using triangular-wave signals at 10, 20, 50, and 100 Hz under both symmetric and asymmetric hysteresis conditions. Figure 12 and Figure 13 show the corresponding experimental results.

As shown in Figure 12 and Figure 13, the hysteresis loops under triangular-wave excitation are also effectively linearized after feedforward compensation. For the symmetric case, the phase compensator substantially reduces the hysteresis-induced deviation, whereas for the asymmetric case, the cascaded phase-compensator–polynomial structure further corrects the residual amplitude asymmetry. The quantitative compensation errors are summarized in Table 2. These results demonstrate that the proposed feedforward compensation method remains effective for the tested non-sinusoidal inputs within the experimentally investigated frequency range of 10–100 Hz.

The slight distortion observed near the turning points of the triangular-wave trajectories is mainly attributed to their broadband harmonic content. A triangular wave consists of a fundamental component and a series of odd harmonics (3f, 5f, 7f, …). At higher excitation frequencies, some of these harmonic components exceed the experimentally investigated compensation range of 10–100 Hz and are consequently subjected to additional amplitude attenuation and phase distortion. Since the sharp corners of a triangular wave depend on these high-frequency harmonics, incomplete compensation of such components leads to the small local deformation near the trajectory reversals.

### 5.4. Experimental Verification of the Proposed Composite Control Strategy

To validate the effectiveness of the proposed composite control strategy, comparative experiments are conducted on a piezoelectric actuator platform. The proposed method employs a phase compensator as the feedforward controller in conjunction with a proportional-integral (PI) feedback loop, while the conventional method adopts a static inverse hysteresis model as the feedforward controller combined with an identical PI feedback structure to ensure a fair comparison. The controller parameters are set to $k_{p}=1$ and $k_{i}=1.2$, and the sampling period is $T_{s}=200$ μs.

For the conventional method, two distinct inverse hysteresis models are established according to the hysteresis characteristics. In both the symmetric and asymmetric cases, the inverse model is constructed based on the Madelung rule-based hysteresis curve description approach [20]. The specific formulations are given as follows. For the symmetric hysteresis case, the major hysteresis curves of the inverse model are expressed as:

(22) $$f_{\mathrm{sym}}^{\prime }\left(x\right)=0.436x^{5}-1.398x^{4}+1.678x^{3}-1.09x^{2}+1.377x,$$

(23) $$g_{\mathrm{sym}}^{\prime }\left(x\right)=-0.2235x^{5}+0.8913x^{4}-1.093x^{3}+0.7319x^{2}+0.6897x.$$

For the asymmetric hysteresis case, the major hysteresis curves of the inverse model are expressed as

(24) $$f_{\mathrm{asym}}^{\prime }\left(x\right)=0.6917x^{5}-2.174x^{4}+2.681x^{3}-1.645x^{2}+1.45x,$$

(25) $$g_{\mathrm{asym}}^{\prime }\left(x\right)=0.1653x^{5}+0.1703x^{4}-0.5709x^{3}+0.6202x^{2}+0.6114x.$$

Two sets of tracking experiments are designed to evaluate the control performance under different hysteresis characteristics, namely symmetric hysteresis tracking and asymmetric hysteresis tracking. In each scenario, the tracking trajectories, tracking errors, and post-compensation hysteresis curves of both methods are recorded and compared. To evaluate the tracking performance over a continuous frequency range, a linear frequency-sweep reference signal is employed. The reference trajectory is defined as

(26) $$y_{d}\left(t\right)=\frac{Y_{\max}}{2}\left\{1-\cos\left[2\pi \left(f_{\mathrm{initial}}t+\frac{f_{\mathrm{target}}-f_{\mathrm{initial}}}{2T_{\mathrm{target}}}t^{2}\right)\right]\right\}.$$

where $Y_{\max}$ denotes the maximum reference displacement. In the experiments, $f_{\mathrm{initial}}=0\;\mathrm{Hz}$, $f_{\mathrm{target}}=60\;\mathrm{Hz}$, and $T_{\mathrm{target}}=10\;s$, corresponding to a sweep rate of 6 Hz/s. The maximum reference displacements are approximately 6.5 μm and 18.5 μm for the symmetric- and asymmetric-hysteresis experiments, respectively.

**Figure 14:**
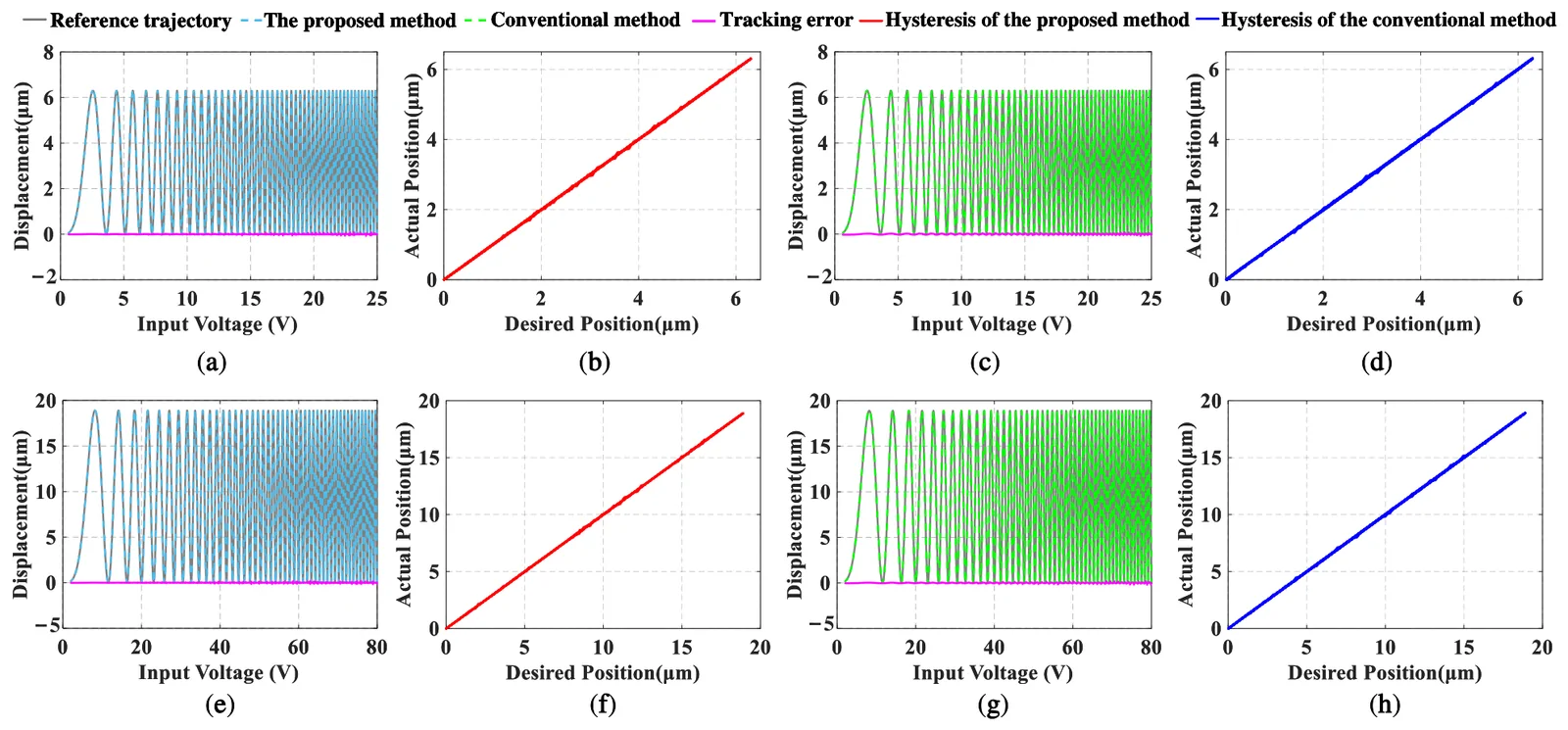
Experimental results of feedback control. Panels (**a**–**d**) correspond to the symmetric hysteresis case, and panels (**e**–**h**) correspond to the asymmetric hysteresis case. Specifically, panels (**a**,**e**) show the tracking trajectories and tracking errors of the proposed method; panels (**b**,**f**) present the corresponding hysteresis curves of the proposed method; panels (**c**,**g**) show the tracking trajectories and tracking errors of the conventional method; and panels (**d**,**h**) present the corresponding hysteresis curves of the conventional method.

The corresponding feedback-control results are shown in Figure 14.

The experimental results indicate that, for both symmetric and asymmetric cases, the compensated hysteresis curves obtained by the two methods exhibit remarkably high linearity, and the tracking errors of the two methods are closely comparable under all tested conditions. Specifically, for symmetric hysteresis, the proposed method achieves an NME of 1.345% versus 1.353% for the conventional method, and an NRMSE of 0.104% versus 0.285%; for asymmetric hysteresis, the proposed method achieves an NME of 0.955% versus 1.110% for the conventional method, and an NRMSE of 0.059% versus 0.150%, as summarized in Table 3. These results confirm that the proposed phase-compensator-based approach effectively circumvents the cumbersome procedure of precise hysteresis modeling and inversion, while achieving control performance that is not inferior to that of the inverse-model-based conventional method, thus offering a practical and efficient alternative for hysteresis compensation in piezoelectric actuators.

## 6. Conclusions

This paper proposes a phase-compensator-based composite control strategy for piezoelectric actuator hysteresis. Unlike conventional model-dependent approaches, the proposed method equivalently treats symmetric hysteresis as linear phase lag and compensates it via a phase compensator. A cascaded polynomial is designed to correct the asymmetric amplitude discrepancy between ascending and descending branches. This hysteresis-model-free scheme avoids cumbersome hysteresis modeling and inversion, featuring a simple structure with few parameters to be identified. Combined with PI feedback control, the composite control achieves tracking errors comparable to inverse-model-based methods. For symmetric hysteresis, the proposed method yields an NME of 1.345% and an NRMSE of 0.104%, versus 1.353% and 0.285% for the conventional method. For asymmetric hysteresis, the proposed method achieves an NME of 0.955% and an NRMSE of 0.059%, compared with 1.110% and 0.150% for the conventional method. Both methods produce compensated hysteresis curves with high linearity. These results demonstrate that the proposed strategy offers a practical and efficient alternative to hysteresis-model-dependent compensation without sacrificing control performance.

## Figures and Tables

**Table 1:** The $e_{\mathrm{NME}}$ and $e_{\mathrm{NRMSE}}$ values of feedforward compensation for the piezoelectric actuator.

		10 Hz	20 Hz	50 Hz	100 Hz
Symmetric hysteresis	$e_{\mathrm{NME}}$	0.68%	1.32%	0.84%	1.48%
	$e_{\mathrm{NRMSE}}$	0.29%	0.55%	0.29%	0.80%
Asymmetric hysteresis	$e_{\mathrm{NME}}$	0.66%	0.72%	0.60%	1.07%
	$e_{\mathrm{NRMSE}}$	0.25%	0.22%	0.29%	0.38%

**Table 2:** Quantitative errors of feedforward compensation under triangular-wave excitation.

		10 Hz	20 Hz	50 Hz	100 Hz
Symmetric hysteresis	$e_{\mathrm{NME}}$	1.12%	1.67%	2.08%	2.57%
	$e_{\mathrm{NRMSE}}$	0.41%	0.86%	1.17%	1.47%
Asymmetric hysteresis	$e_{\mathrm{NME}}$	2.05%	2.69%	2.72%	2.95%
	$e_{\mathrm{NRMSE}}$	1.11%	1.57%	1.59%	1.73%

**Table 3:** The $e_{\mathrm{NME}}$ and $e_{\mathrm{NRMSE}}$ values of feedback control for the piezoelectric actuator.

		Proposed Method	Conventional Method
Symmetric hysteresis	$e_{\mathrm{NME}}$	1.345%	1.353%
	$e_{\mathrm{NRMSE}}$	0.104%	0.285%
Asymmetric hysteresis	$e_{\mathrm{NME}}$	0.955%	1.110%
	$e_{\mathrm{NRMSE}}$	0.059%	0.150%

## Data Availability

All data generated or analysed during this study are included in this published article.

## References

[1] Sun J., Deng J., Li J., Zhang S., Xun M., Liu Y. (2026). Development of a stick-slip piezoelectric screw actuator with high thrust force density. IEEE Trans. Ind. Electron..

[2] Yu H., Liu Y., Deng J., Zhang S., Chen W. (2022). A collaborative excitation method for piezoelectric stick-slip actuator to eliminate rollback and generate precise smooth motion. Mech. Syst. Signal Process..

[3] Hao G., Cao K., Li R., Li Z., Du H., Tan L. (2024). Rate-dependent hysteresis modeling and compensation for fast steering mirrors. Sens. Actuators A Phys..

[4] Cao K., Li Z., Hao G., Li R., Zhang J., Ma J. (2025). Modified hammerstein-like hysteresis modeling and composite control methods for fast steering mirrors. Micromachines.

[5] Hao G., Cao K., Li Z., Du H., Tan L. (2026). A composite control approach and its simplified form for piezoelectric ceramic-driven fast steering mirrors: From model-based to model-free. Sens. Actuators A Phys..

[6] Cao K., Li R. (2019). Modeling of Rate-Independent and Symmetric Hysteresis Based on Madelung’s Rules. Sensors.

[7] Gu G.-Y., Zhu L.-M., Su C.-Y., Ding H., Fatikow S. (2016). Modeling and control of piezo-actuated nanopositioning stages: A survey. IEEE Trans. Autom. Sci. Eng..

[8] Zou J., Gu G. (2019). High-precision tracking control of a soft dielectric elastomer actuator with inverse viscoelastic hysteresis compensation. IEEE/ASME Trans. Mechatron..

[9] Li Z., Fan X., Long D., Yang Z., Shi C. (2025). A modular direct description method with low memory usage and execution time for hysteresis modeling and compensation of piezoelectric actuators. IEEE Trans. Autom. Sci. Eng..

[10] Yuan Z., Li X., Chen X., Wu J. (2026). A novel rate-dependent hysteresis framework for piezoelectric actuators: Modeling, identification, and control. IEEE Trans. Ind. Electron..

[11] Zhang C., Yu Y., Zhou M. (2023). Finite-time adaptive quantized motion control for hysteretic systems with application to piezoelectric-driven micropositioning stage. IEEE/ASME Trans. Mechatron..

[12] Li Z., Shan J., Gabbert U. (2021). A direct inverse model for hysteresis compensation. IEEE Trans. Ind. Electron..

[13] Nie L., Luo Y., Gao W., Zhou M. (2022). Rate-dependent asymmetric hysteresis modeling and robust adaptive trajectory tracking for piezoelectric micropositioning stages. Nonlinear Dyn..

[14] Liu Y., Du D., Qi N., Zhao J. (2019). A distributed parameter maxwell-slip model for the hysteresis in piezoelectric actuators. IEEE Trans. Ind. Electron..

[15] Al Janaideh M., Krejčí P. (2013). Inverse rate-dependent prandtl-ishlinskii model for feedforward compensation of hysteresis in a piezomicropositioning actuator. IEEE/ASME Trans. Mechatron..

[16] Yi S., Zhang Q., Xu L., Wang T., Li L. (2022). Hysteresis online identification approach for smart material actuators with different input signals and external disturbances. Nonlinear Dyn..

[17] Zhang H.T., Hu B., Li L., Chen Z., Wu D., Xu B., Huang X., Gu G., Yuan Y. (2018). Distributed hammerstein modeling for cross-coupling effect of multiaxis piezoelectric micropositioning stages. IEEE/ASME Trans. Mechatron..

[18] Su L., Gao W., Zhang X., Su C.-Y., Zhou M. (2026). Event-triggered sliding mode predictive control for piezoelectric-actuated hybrid vibration isolators subject to hysteresis and time delays. Mech. Syst. Signal Process..

[19] Nie L., Yu Y., Zhou M., Zhang X., Su C.-Y. (2025). Hysteresis-estimator-based adaptive fuzzy control for piezoelectric micro-positioning stage with time-varying output constraints. IEEE Trans. Circuits Syst. II Express Briefs.

[20] Li R., Cao K., Yu X., Zeng M. (2021). Modeling and compensation algorithms of asymmetric nonlinearity for piezoelectric actuators based on madelung’s rules. IEEE Trans. Ind. Electron..

[21] Gu G.-Y., Zhu L.-M., Su C.-Y. (2014). Modeling and compensation of asymmetric hysteresis nonlinearity for piezoceramic actuators with a modified Prandtl–Ishlinskii model. IEEE Trans. Ind. Electron..

[22] Wang G., Chen G., Bai F. (2015). Modeling and identification of asymmetric Bouc–Wen hysteresis for piezoelectric actuator via a novel differential evolution algorithm. Sens. Actuators A Phys..

[23] Wang W., Wang J., Chen Z., Wang R., Lu K., Sang Z., Ju B. (2020). Research on asymmetric hysteresis modeling and compensation of piezoelectric actuators with PMPI model. Micromachines.

